# Case Report: Two Patients With EGFR Exon 20 Insertion Mutanted Non-Small Cell Lung Cancer Precision Treatment Using Patient-Derived Xenografts in Zebrafish Embryos

**DOI:** 10.3389/fonc.2022.884798

**Published:** 2022-07-20

**Authors:** Qian Wang, Wenxian Wang, Weiwei Pan, Xiaojing Lv, Lei Zhang, Kaiming Zheng, Fang Tian, Chunwei Xu

**Affiliations:** ^1^ Department of Respiratory Medicine, Affiliated Hospital of Nanjing University of Chinese Medicine, Jiangsu Province Hospital of Chinese Medicine, Nanjing, China; ^2^ Department of Respiratory Medicine, Affiliated Hospital of Nanjing University of Chinese Medicine, Suqian Hospital of Chinese Medicine, Suqian, China; ^3^ Department of Medical Oncology, The Cancer Hospital of the University of Chinese Academy of Sciences (Zhejiang Cancer Hospital), Hangzhou, China; ^4^ Department of Cell Biology, College of Medicine, Jiaxing University, Jiaxing, China; ^5^ Department of Geriatric Medicine, Affiliated Hospital of Nanjing University of Chinese Medicine, Jiangsu Province Hospital of Chinese Medicine, Nanjing, China; ^6^ Department of Respiratory Medicine, Jinling Hospital, Nanjing University School of Medicine, Nanjing, China

**Keywords:** non-small cell lung cancer, zebrafish, EGFR mutation, xenograft, screening

## Abstract

**Background:**

Epidermal growth factor receptor (EGFR) exon 20 insertion mutations are uncommon EGFR mutations and generally resistant to first- and second-generation EGFR-tyrosine kinase inhibitors (TKIs). In precision oncology, treatment regimens are tested for improving the clinical outcomes. Zebrafish embryo tumor transplant models are used in cancer research.

**Methods:**

We report two Chinese females who were diagnosed with stage IV lung adenocarcinoma and shown to harbor EGFR exon 20 insertion mutations by next-generation sequencing (NGS). Then, we established lung cancer patient-derived xenografts using a zebrafish model. The tumor cells were isolated from the patient. For case one, tumor cells were collected from lymph node biopsy, while the tumor cells were obtained from the pleural effusion. Zebrafish were inoculated with tumor cells and placed in the culture medium containing the third-generation EGFR-TKI, osimertinib. Fluorescence microscope photographs were used to record the red fluorescence area, which represented the proliferation and migration of tumor cells in the zebrafish.

**Results:**

Case one was diagnosed with lung adenocarcinoma (cT4N3M1b, stage IVB) and had an EGFR exon 20 mutation (p. N771delinsHH [abundance 14.08%]). Tumor cell proliferation and migration were significantly reduced in the osimertinib group compared with the control group. The patient received first-line osimertinib (160 mg). According to RECIST v1.1, she achieved a partial response. Case two had stage IVA lung adenocarcinoma with a pleural effusion. The pleural effusion sample was selected to obtain tumor cells for injection, and the zebrafish lung cancer model was established. The proliferation of tumor cells in the osimertinib group was significantly reduced compared to the control group. The migration of tumor cells was not significantly reduced compared to the control group. The patient also received first-line osimertinib (160 mg). The lung lesions were stable, but the pleural effusion was poorly controlled.

**Conclusion:**

Our study demonstrates the applicability of a zebrafish embryos model as an innovative platform to targeted drug testing. More precise methods are needed to select treatment options in the future.

## 1 Introduction

The emergence of targetable oncogenic driver alterations has transformed treatment models of non-small cell lung cancer (NSCLC) by incorporating tumor genotyping into therapeutic strategies. Specifically, epidermal growth factor receptor (EGFR)-activating mutations have resulted in routine use of EGFR tyrosine kinase inhibitors (TKIs) (1, 2). EGFR mutations mainly occur between exons 18 and 21 in NSCLC; common mutations are an EGFR 19 deletion and EGFR mutations in exon 21. Drugs against cancers harboring common EGFR mutations have a response rate of 60%–70%, with a median progression-free survival (mPFS) of 9.2–18.9 months (1, 3–6).

An EGFR exon 20 insertion mutation is an uncommon subtype of EGFR mutation that accounts for 4%–10% of all EGFR mutations (6). Exon 20 insertion mutations include A767-V769dup and D770-N771ins NPG, which are also associated with a lack of sensitivity to first- or second-generation EGFR-TKIs (6, 7). Therefore, the standard treatment for patients with EGFR exon 20 insertions is a chemotherapy-based treatment regimen (8). The development of novel targeted drugs, such as poziotinib (9), mobocertinib (TAK-788) (10), and amivantamab (JNJ-61186372) (11), have shown better efficacy in the treatment of EGFR exon 20 insertion mutations. Poziotinib, is an irreversible pan-HER TKI, initially being investigated in the Asian population, shown a slightly better ORR/DCR and PFS in EGFR exon 20 insertion mutations. Mobocertinib inhibited viability of various EGFRex20ins-driven cell lines more potently than approved EGFR TKIs and demonstrated *in vivo* antitumor efficacy in patient-derived xenografts and murine orthotopic models. Amivantamab inhibited proliferation by effectively downmodulating EGFR-MET levels and inducing immune-directed antitumor activity with increased IFN γ secretion in various models. However, these targeted drugs are not currently available in China.

Osimertinib is an irreversible, selective EGFR TKI that is indicated for sensitizing EGFR and EGFR T790M resistance mutations. Preclinical studies have reported that osimertinib is active in EGFR exon 20 insertion mutant cell lines (12, 13) and some clinical trials have also demonstrated clinical activity in EGFR 20 insertion mutant NSCLC (14–16); however, *in vitro* evidence demonstrated 20 insertion mutation cell lines that responded poorly to osimertinib (12, 13, 17). Several studies have reported that the overall response rate (ORR) and progression-free survival (PFS) differed among patients with 20 insertion mutations (15, 16, 18).

Currently, patient-derived xenografts (PDXs) or patient-derived organoids (PDOs) are used in tumor preclinical models in which the genetic mutation map of tumor characteristics is highly consistent with the original tumor tissue with respect to morphology and genetic characteristics (19, 20). Tumor cell behavior in zebrafish xenografts correlates with human cancers as follows: similar growth kinetics; histology; and proliferation and apoptosis rates (21). Herein, to give patients more precision treatment options, we studied two NSCLC patients with EGFR exon 20 insertion mutations who were the source of PDXs used in zebrafish embryos as *in vitro* tumor models for therapeutic screening. In addition, we assessed the antitumor activity of osimertinib in the two NSCLC patients with EGFR exon 20 insertion mutations.

## 2 Methods

### 2.1 Lung Cancer PDXs Using a Zebrafish Model

In this model, 48 hpf AB wild-type zebrafish embryos were selected for microinjection of tumor cells, with approximately 800 cells/embryo, to establish a PDX model of zebrafish lung cancer. The 48 hpf AB wild-type zebrafish embryos were obtained from the Model Animal Research Center of Nanjing University (Nanjing, China). The zebrafish embryos were maintained at 28.5°C in a 14:10 h light/dark cycle. Embryos were obtained by mixing two males and two females in a water tank equipped with a grill to avoid the introduction of new eggs. The fish were mated and spawned at the beginning of the light period. Embryos were collected and placed in petri dishes containing an embryo medium (0.2 g/L of Instant Ocean^®^ Salt in distilled water) at 28.5°C. Whole embryos were pooled and counted, and the malformed embryos were discarded. The age of the embryos is represented by hours post-fertilization (HPF). After removal of the chorionic villi, the embryos were immersed in an embryo medium containing 0.2 mM 1-phenyl-2-thiourea after 24h of incubation at 28.5°C. At 48 HPF, the embryos were anesthetized with 0.0003% tetracaine (Sigma-Aldrich) and placed on a wet agarose pad with the right side up. Approximately 200 cells were injected into the yolk sac of the embryos using a microinjector (im-31; Narishige, Japan). The zebrafish were placed in an incubator at 28°C for 3 h. The zebrafish that were successfully transplanted with tumor cells and relatively uniform in size were selected using a stereoscopic fluorescence microscope for follow-up observation of tumor migration and angiogenesis. At least 10 fish were subjected to each treatment. The images of the sub-intestinal venous plexus (SIV) were obtained using a Leica MZ10 F fluorescence microscope. For tumor proliferation, a group of 10 embryos was selected and dissociated into a single cell suspension.

One day after tumor cell inoculation (1 dpi), zebrafish embryos of uniform tumor size were screened and randomly divided into control and experimental groups (soaked drugs [grouped by different drugs]). Zebrafish inoculated with tumor cells for 1 day (1 dpi) were placed in the culture medium containing drugs, and the fresh culture medium containing drugs was replaced every 24 h for 3 consecutive days. The red fluorescence area (representing zebrafish with proliferation and migration of tumor cells in the body) were recorded in control and experimental groups with fluorescence microscope photos after 3 consecutive days.

### 2.2 Follow-Up Data

The patients were followed by one year or until death.

### 2.3 DNA Extraction and Next-Generation Sequencing

Two paraffin blocks of formalin fixed tissue or mass cells were collected from department of pathology from Affiliated Hospital of Nanjing University of Chinese Medicine. The DNA extraction and the next generation sequencing was conducted by Burning Rock Company (Guangzhou, China).

### 2.4 Image and Data Analysis

Image J software was used for image processing. Graphpad Prism 8 software was used for statistical analysis. All statistical tests were two-sided, and a P value <0.05 was considered statistically significant.

## 3 Case Report

The study was approved by the Institutional Review Board of Jiangsu Province Hospital of Chinese Medicine and by the Ethics Committee of Jiangsu Province Hospital of Chinese Medicine. Informed consent was obtained from both patients.

### 3.1 Case One

**Figure 1:**
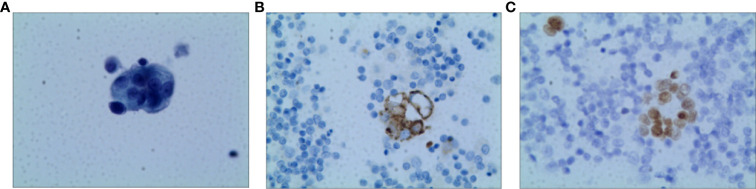
Pathology of Case one patient **(A)** H&E staining suggested lung adenocarcinoma (X400) **(B)** The result of IHC-Napsin A was positive (X400). **(C)** The result of IHC-TTF-1 was positive (X400).

A 73-year-old non-smoking female was admitted to the hospital in September 2020 for evaluation of a cough. A computed tomography (CT) scan showed a right middle lobe mass, multiple mediastinal lymph node metastases, metastatic supraclavicular lymph nodes, multiple solid nodules in both lungs with bilateral pleural effusions, and a pericardial effusion. A brain MRI showed no metastases and bone imaging revealed bone destruction in the third thoracic vertebra with bone metastases. Cytology of the pleural fluid showed a profiled epithelial cell mass that was suspected to be adenocarcinoma. A puncture biopsy of the right mediastinal lymph node suggested lung adenocarcinoma (**Figure 1**). The patient was subsequently diagnosed with lung adenocarcinoma (cT4N3M1b, stage IVB). DNA was extracted from the cell block of the pleural effusion for next-generation sequencing (NGS). Genetic testing showed that the patient had an EGFR exon 20 mutation (p.N771delinsHH [abundance 14.08%]).

**Figure 2:**
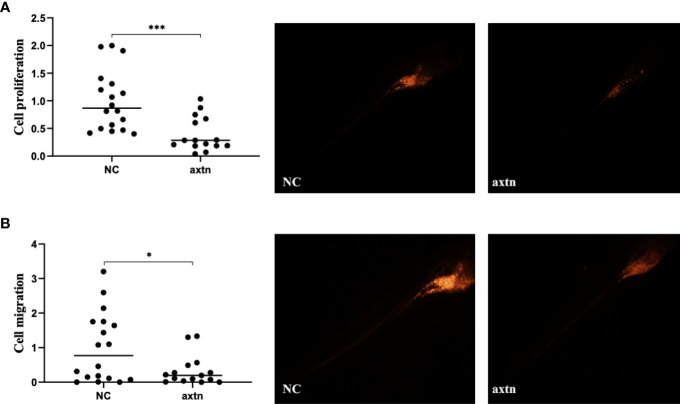
Zebrafish lung cancer model was established **(A)** Tumor cell proliferation was significantly reduced in the osimertinib group compared with the control group **(B)** Migration were significantly reduced in the osimertinib group compared with the control group. Whole-body image of the zebrafish embryo at 4 dpi (25× magnification). *(P<0.05), ***(P<0.001).

A lymph node biopsy tissue sample of the patient was selected to obtain tumor cells for injection. A zebrafish lung cancer model was established with the above method for therapeutic screening. Tumor cell proliferation and migration were significantly reduced in the osimertinib group compared with the control group (**Figures 2A, B**).

**Figure 3:**
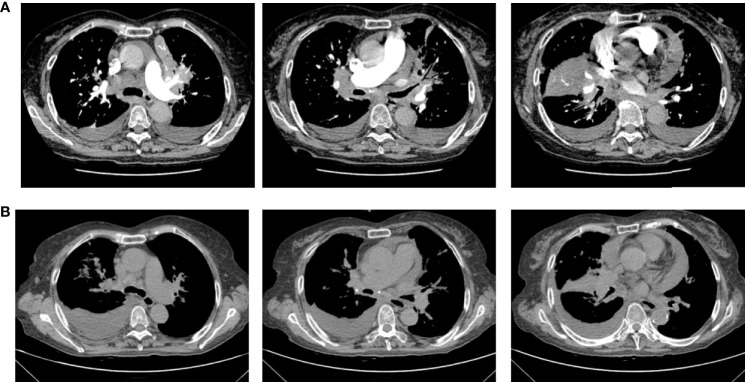
Chest computerized tomography (CT) scan images of patient one before and after one month of osimertinib treatment. **(A)** The images were before osimertinib treatment. **(B)** The images of were receiving osimertinib treatment after one month.

Therefore, due to the patient’s advanced age and PS score of 3, chemotherapy was not acceptable. The patient received first-line osimertinib (160 mg) in October. According to RECIST v1.1, she achieved a partial response (**Figure 3**) after 1 month of treatment, then unfortunately developed interstitial pneumonia. She discontinued osimertinib and received steroid treatment. After 2 weeks, a CT showed interstitial pneumonia that progressed in December. Beginning in December 2020 the patient received anlotinib as second-line treatment; however, the disease progressed and she died on 31 December 2020.

### 3.2 Case Two

**Figure 4:**
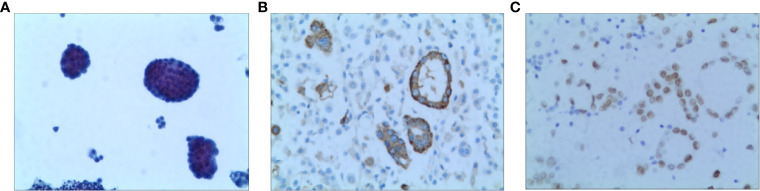
Pathology of Case two patient **(A)** H&E staining suggested lung adenocarcinoma (X100) **(B)** The result of IHC-Napsin A was positive (X400). **(C)** The result of IHC-TTF-1 was positive (X400).

A 52-year-old non-smoking female was admitted to the hospital in October 2020 due to chest pain that persisted > 1 month. A CT scan showed that the right lung had bilateral pleural effusions and pulmonary atelectasis. A brain MRI and bone imaging showed no metastases. Cytologic evaluation of the pleural fluid was consistent with lung adenocarcinoma (**Figure 4**). The patient was diagnosed with lung adenocarcinoma (cT4N0M1a, stage IVA). DNA was extracted from the cell block of pleural effusion for NGS. Genetic testing showed the patient had an EGFR exon 20 mutation (p.H773_V774insPHPH).

**Figure 5:**
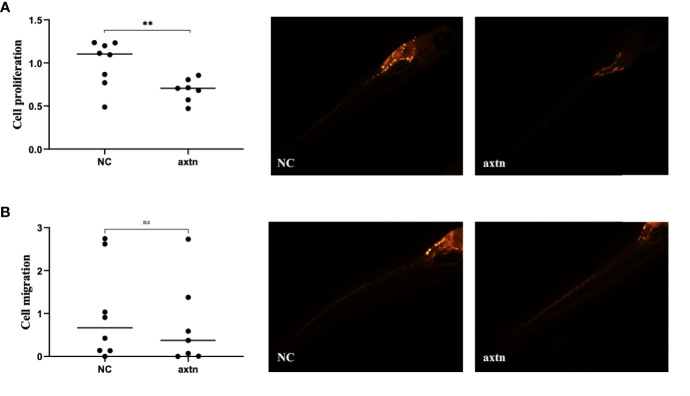
Zebrafish lung cancer model was established **(A)** The proliferation of tumor cells were significantly reduced in the osimertinib group compared with the control group **(B)** The migration of tumor cells was not significantly reduced compared with the control group. Whole-body image of the zebrafish embryo at 4 dpi (25× magnification). **(P<0.01), ns: no significance.

A pleural effusion sample from the patient was selected to obtain tumor cells for injection, and the zebrafish lung cancer model was established with the above method for therapeutic screening. The proliferation of tumor cells in the osimertinib group was significantly reduced compared with the control group, and the difference was statistically significant (**Figure 5A**). The migration of tumor cells in the osimertinib group was not significantly reduced compared with the control group (**Figure 5B**), suggesting that osimertinib could be used as a treatment choice for this patient.

**Figure 6:**
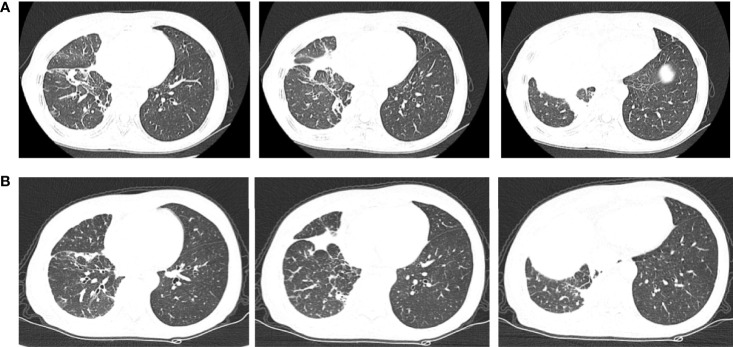
Chest computerized tomography (CT) scan images of patient two before and after one month of osimertinib treatment. **(A)** The images were before osimertinib treatment. **(B)** The images were receiving osimertinib treatment after one month.

Chemotherapy was not acceptable, thus the patient received first-line osimertinib (160 mg) in October. According to RECIST v1.1, she achieved stable disease (**Figure 6**) and the pleural effusion was not controlled. The pleural fluid was repeatedly drained. Osimertinib treatment was continued for 3 months.

## 4 Discussion

The results of these two cases indicated that third-generation EGFR-TKIs could be used as a therapeutic choice for patients with an EGFR 20 exon insertion mutation, and patient-derived zebrafish embryo xenotransplantation was used as an *in vitro* tumor model for therapeutic screening with accurate prediction and guidance for precise clinical treatment.

Innate immunity in zebrafish plays a key role in engraftment success of implanted cancer cells that is cell line-dependent (22). Currently, > 50 zebrafish models of human cancer have been established in which the histologic and/or genomic levels were closely human counterparts (23). Zebrafish in cancer models have promoted the exploration of new mechanisms and identified new drugs (24). The zebrafish embryos have a short generation time and the transparency which enables non-invasive imaging could facilitate visualization of tumor cell behavior (25, 26). Recently, research illustrated that the zebrafish tumor xenograft platform provide a fast, accurate, and clinically relevant system for evaluation of treatment outcome and invasion/dissemination of PDX models, providing an attractive platform for combined mouse-zebrafish PDX trials and personalized medicine (27). In the current study we collected pleural effusion samples from both patients with lung adenocarcinoma, which successfully established the zebrafish embryo PDX model. This method greatly reduces the time of establishing the PDX model and has the advantage of more rapid screening of effective treatment in the clinic setting.

NSCLC with EGFR exon 20 insertion mutations represent a unique subset of advanced NSCLC patients, in whom target therapy has demonstrated little efficacy and the standard first-line therapy is the same as EGFR-negative patients (28). In recent years, targeted therapies for EGFR 20 insertion mutations have been explored; however, because of the availability of the drugs and the demonstration in several preclinical studies that osimertinib is active in cell lines with EGFR exon 20 insertion mutations, osimertinib is a preferred treatment option. In some studies, the efficacy of osimertinib has been controversial. Fang et al. (15) reported a mPFS of 6.2 months in 6 Chinese EGFR exon 20 insertion-mutated NSCLC patients. Although there are some studies addressing the poor activity of osimertinib for EGFR 20 insertion mutations. Yang and colleagues (28) showed that the mPFS in 62 patients was 2.3 months and the A763_Y764insFQEA and D770delinsGY might respond better to osimertinib than the other exon 20 insertion subtypes (18). Therefore, we reasoned that different EGFR 20 insertion mutation subtypes have different efficacies for osimertinib treatment. We are of the opinion that the PDX model is a promising platform to perform preclinical drug screening. In case one, drug screening *in vitro* showed that osimertinib significantly inhibited the proliferation and migration of tumor cells. The tumor was significantly reduced and anastomosed after treatment. In case two, drug screening *in vitro* showed that osimertinib inhibited the proliferation of tumor cells, but had no significant effect on migration. The primary tumor of the patient was well-controlled after osimertinib treatment, however, metastatic lesions were not well-controlled, which was consistent with the results of a zebrafish model. Moreover, zebrafish can be used to demonstrate the heterogeneity of tumors. Therefore, two patients received precision treatment through PDXs in zebrafish embryos as *in vitro* cancer models for therapeutic screening.

## Conclusions

In conclusion, we showed the applicability of PDXs in zebrafish embryos model as an innovative platform to targeted drug screening. Our future studies with a larger sample size will focus on more accurate localization of beneficial populations of NSCLC.

## Data Availability Statement

The original contributions presented in the study are included in the article/supplementary material. Further inquiries can be directed to the corresponding authors.

## Ethics Statement

Written informed consent was obtained from the participant for the publication of this case report.

## Author Contributions

All authors contributed to the article and approved the submitted version.

## Funding

This study was supported in part by grants from National Natural Science Foundation of China (81873277, 31871402), Zhejiang Tranditional Chinese Medicine Science Fund Project (2021ZQ013), Huilan Public-Hanson Pharmaceutical Lung Cancer Precision Medical Research Special Fund Project Foundation (HL-HS2020-5) and Xisike-Hanson Cancer Research Foundation (Y-HS2019-20).

## Conflict of Interest

The authors declare that the research was conducted in the absence of any commercial or financial relationships that could be construed as a potential conflict of interest.

## Publisher’s Note

All claims expressed in this article are solely those of the authors and do not necessarily represent those of their affiliated organizations, or those of the publisher, the editors and the reviewers. Any product that may be evaluated in this article, or claim that may be made by its manufacturer, is not guaranteed or endorsed by the publisher.
